# Beam Steering 3D Printed Dielectric Lens Antennas for Millimeter-Wave and 5G Applications

**DOI:** 10.3390/s23156961

**Published:** 2023-08-05

**Authors:** Asrin Piroutiniya, Mohamad Hosein Rasekhmanesh, José Luis Masa-Campos, Javier López-Hernández, Eduardo García-Marín, Adrián Tamayo-Domínguez, Pablo Sánchez-Olivares, Jorge A. Ruiz-Cruz

**Affiliations:** 1 Group of RadioFrequency: Circuits and Systems (RFCAS), Escuela Politécnica Superior, Universidad Autónoma de Madrid, 28049 Madrid, Spain; asrin.piroutiniya@estudiante.uam.es (A.P.); mohamad.rasekhmanesh@estudiante.uam.es (M.H.R.); javier.lopezh@estudiante.uam.es (J.L.-H.); eduardo.garciam@uam.es (E.G.-M.); jorge.ruizcruz@uam.es (J.A.R.-C.); 2 Centro de Investigación en Procesado de la Información y Telecomunicaciones, Escuela Técnica Superior de Ingenieros de Telecomunicación (ETSIT), Universidad Politécnica de Madrid, 28040 Madrid, Spain; a.tamayo@upm.es (A.T.-D.); pablo.sanchezo@upm.es (P.S.-O.)

**Keywords:** lens antenna, 3D printing, manufacturing by stereolithography, pencil beam, 5G, horn antenna, millimeter-wave lens

## Abstract

Two types of cost-efficient antennas based on dielectric gradient index dielectric lens have been designed for 5G applications at 28 GHz. The first is a linearly polarized flat lens antenna (LP-FLA) for terrestrial 5G communications. The second is a novel circularly polarized stepped lens antenna (CP-SLA) for 5G satellite services. An efficient design method is presented to optimize and conform the lens topology to the radiation pattern coming from the antenna feeder. The LP-FLA is fed by a traditional linearly polarized pyramidal horn antenna (PHA). The CP-SLA is fed by an open-ended bow-tie waveguide cavity (BCA) antenna. This cavity feeder (BCA), using cross-sections with bow-tie shapes, allows having circular polarization at the desired frequency bandwidth. The two types of presented antennas have been manufactured in order to verify their performance by an easy, low-cost, three-dimensional (3D) printing technique based on stereolithography. The peak realized gain value for the flat (LP-FLA) and stepped (CP-SLA) lens antennas have been increased at 28 GHz to 25.2 and 24.8 dBi, respectively, by disposing the lens structures at the appropriated distance from the feeders. Likewise, using an array of horns (PHA) or open-ended bow-tie waveguide cavity (BCA) antenna feeders, it is possible to obtain a maximum steering angle range of 20° and 35°, for a directivity over 15 dBi and 10 dBi, in the planar and stepped lens antennas, respectively.

## 1. Introduction

Multi-beam antennas (MBAs) are defined as antennas that generate either multiple numbers of beams simultaneously, or switch between beams in multiple directions. MBAs have some advantages such as the ability to focus the radiation on a desired direction, to increase the capacity of the wireless system, and to facilitate tracking objects in radar systems [1]. MBAs with linear and circular polarization can be used for base-station applications and satellite communications [2,3]. The antenna directivity must be high enough to overcome propagation losses, having very low side lobes and a main lobe concentrating most of the radiated energy. This feature leads to a pencil shape pattern. Several feeders with different inputs in different positions can be placed to have multi-beam functions. Each feeder generates a specific beam in a different direction, which enable the entire system to cover a wide area range. MBAs have been widely employed in satellite communications and radar systems by providing highly directive pencil beams. On the other hand, the fifth-generation standard (5G) brings novel concepts to use MBAs because of their ability to produce multiple parallel, but independent, sharp beams with a high gain value to cover a given angular range. In addition, MBAs for base stations with fan-shaped beams are a key element in terrestrial communications due to their gain and reduced interferences [3,4,5,6].

Multiple-input, multiple-output (MIMO) is an antenna technology that employs multiple arrays of antennas at both the transmitter and the receiver. The antenna signals at each end of the communication circuit are combined with different coefficients to achieve the best overall antenna gain. Thus, they require a signal processing stage after the arrays. The MIMO system creates a more stable connection with less congestion. A wide range of applications utilize MIMO technology, including Wi-Fi networks, Long Term Evolution (LTE) and fifth-generation (5G) cellular technologies. Lens antennas can also be found in this context, which can be used for multi-beam performance [7]. Due to its use in massive upcoming 5G applications, MIMO is evolving and growing by using numerous small antennas to boost bandwidth for users. In fact, massive MIMO technology provides high beam forming gain, which leads to better coverage. It also reduces the interference as the MIMO beam width becomes narrower.

Additionally, because 5G requires a high data transmission speed [5], 5G antennas can be employed for millimeter-wave applications. Increasing the absolute bandwidth and employing higher frequencies are two ways for obtaining higher data rates. However, the free-space propagation losses increase at higher frequencies, requiring a higher gain. Therefore, millimeter-wave antennas are currently attracting a lot of attention for different applications. The millimeter-wave band provides significantly more bandwidth resources than lower frequency bands, paving the way for the upcoming 5G and 6G networks with high capacity [8]. A high-speed millimeter-wave MIMO communication system has been presented in [8], which developed a testbed combining millimeter-wave and MIMO for improving transmission rate and sensing accuracy. Work in [7] provides a unique integrated sensing and communication strategy for huge MIMO systems that employs the joint beam-squint and beam-split effect.

In [9], a special category of passive beam steering antennas based on lenses is presented. Lens antennas can be used with 5G satellites and base stations to transmit voices and data. They have a high gain that allows covering a wide area and great distances [3]. Lens antennas are well-known for shaped beam applications and gain enhancement because of their ability to focus electromagnetic waves on certain directions. The cost of manufacturing also contributes significantly to the superiority of these antennas. The three-dimensional (3D) printing method can be a great alternative to further reduce the fabrication cost. Manufacturing complex geometries easily, low-priced, rapid and high accuracy, are the characteristics of the 3D printing method, which can be exploited to fabricate horns [10] and lens antennas [11].

Lens antennas can use multiple kind of feeders located at its focal distance: patches [12], horns [13,14] or even array antennas [15]. Besides the faculty to efficiently obtain directive patterns with a simple design, it is also apparently easy to extend this functionality to generate several radiation beams with different pointing angles. In this sense, ref. [16] shows how a simple dielectric lens can increase the maximum gain of a microstrip patch antenna to 16 dBi at 60 GHz. A similar structure of the dielectric lens, which is placed in front of a horn antenna, is introduced in [17], increasing the maximum gain up to 21 dBi at 60 GHz. Furthermore, a mechanical structure allows the dielectric lens to rotate, enabling a compact beam-steerable lens antenna for wireless communications. Several recent studies have demonstrated the potential benefits of 3D printing for the design of high-performance lens antennas [18,19]. Antenna components can be manufactured based on arbitrary 3D structures thanks to the high-resolution printing technology and flexible fabrication process.

Gradient index (GRIN) materials, as well as their use in lens antennas, have been the subject of a great area of research over the past few years. Electromagnetic structures with a gradual index of refraction are called gradient index components. For perforated dielectrics with through-hole cylindrical perforations, work in [20] provides a straightforward analytical effective permittivity model. In this case, various devices widely used in lens antenna applications rely on GRIN materials, such as the Luneburg lens [21], Maxwell fisheye lens [22], and dielectric flat lens [4,23].

In this paper, a design process for perforated GRIN lens antennas is described. The theoretical match between the feeder radiation pattern and the lens physical size is shown in detail to maximize the overall antenna efficiency. To confirm the viability of the proposed technique, two distinct perforated GRIN lens antennas are also provided. In one case, a flat perforated GRIN lens is designed for the new high-data rate and low latency 5G communications, for terrestrial applications at 28 GHz. A pyramidal linearly polarized horn antenna feeder is optimally assembled to the lens structure in order to increase the global antenna efficiency. The second concept, suitable for 5G satellite applications at 28 GHz, is a novel stepped perforated GRIN lens fed by a bow-tie cavity [24] to achieve circular polarization.

The work will be covered in three main sections. In the first one, corresponding to Section 2, the GRIN lens antenna design procedure is detailed. It will be especially focused on the optimization of the antenna total aperture efficiency as a function of the electric field amplitude of the feeder radiation pattern, the focal distance of the structure, and the lens diameter. The optimum design conditions are later established, directly related to these antenna parameters. Likewise, the phase optimization of the total electric field at the antenna aperture is also explained. The goal is to achieve the optimum performance with the use of a GRIN lens, which is finally implemented in perforated topology. In Section 3 and Section 4, two different types of dielectric GRIN lens antennas with different feeders are discussed for 5G communications at 28 GHz. One is a linear polarized flat lens antenna (LP-FLA) for terrestrial applications in Section 3. The second is a novel concept of circular polarized stepped lens antenna (CP-SLA) for satellite systems in Section 4. This achieves an improved performance in terms of phase uniformity of the electric field at the aperture, with a volume reduction of the global antenna structure.

A circularly polarized bowtie cavity antenna (BCA) feeds the CP-SLA, while a linearly polarized pyramidal horn antenna (PHA) feeds the LP-FLA. Both concepts employ perforated dielectrics with through-hole cylindrical perforations, that is, holes arranged following a certain pattern. The provided rings implement different effective dielectric constants ($\varepsilon_{r}$) to achieve a uniform phase of the propagated wave. The propagated wave from the feeder arrives at the lens structure via various paths, resulting in different phase values. By modifying the dielectric constant, a uniform phase of the electric field at the aperture can be achieved, resulting in maximum efficiency and directivity. A representative prototype has been manufactured for both lens antenna concepts, using 3D printing based on stereolithography. In the case of the antenna feeders (BCA and PHA), a copper coating method [25] has been later applied to metallize the fabricated plastic modules. The validation process with electrical measurements and simulation comparatives is also presented for each one of the two antenna prototypes.

Several novelties have been introduced in this work. The first novelty is the design strategy used in these lens antennas to obtain a high aperture efficiency. In the presented approach, the interaction between the lens parameters (mainly the focal distance *F* and the lens diameter *D*) and the required specifications for the lens feeder, are crucial for adequately illuminating the lens edges at the proper level. This approach allows achieving optimum high aperture efficiency by establishing the necessary feeder radiation properties. To validate the idea, two different designs are introduced at the millimeter-wave band.

The first design is a flat lens antenna (FLA) fed by a pyramidal horn antenna (PHA) with linear polarization. The proposed design method leads to a high antenna performance. Only a single novelty is introduced in this structure aside from the method validation. This novelty is the concentric arrangement for the ring holes, in comparison to the triangular pattern in the state of the art. It will be explained in detail in Section 2.4 that, in the triangular arrangement, every ring occupies more space to ensure the dielectric constant equivalent value. Thus, less samples can be implemented in the ring area. In the concentric ring arrangement, as every ring is thinner, more samples in the dielectric constants can be implemented. However, there is a risk of not having the exact desired value on every one of them.

The second design and highlighted novelty is the stepped lens antenna (SLA), which has been fed by a circular polarized bow-tie cavity antenna (BCA). This design avoids the 2π phase turns for improving the phase uniformity of the electric field at the aperture in the design frequency range (detailed in Section 2.2). Furthermore, the focal distance for the same antenna aperture efficiency has been reduced. The approach allows several *F*/*D* ratios for the same optimum design and implementing the closest *F* distance. This leads to a wider beam width of the antenna feeder with a more compact system (explained in detail in Section 2.2).

## 2. GRIN Lens Antenna Design Procedure

A graded index lens (GRIN) is a type of lens antenna in which the dielectric permittivity is radially modified to properly focus the radiated wave from a feeder antenna, with a consequent gain increase. Due to the similarities between lens antennas and reflector-based antennas, the total aperture efficiency ($\eta_{Total-aper}$) should be taken into consideration during the design process as follows:

(1) $$\eta_{Total-aper}=\eta_{aper}\;\cdot \;\eta_{pol\;}\cdot \;\eta_{block}\;\cdot \;\eta_{error}$$

Equation (1) shows that there are several different contributions to this efficiency. Aperture efficiency $\;(\eta_{aper})$ simultaneously evaluates the uniformity of the illuminated electric field (E-field from now on) at the lens aperture and the wasted E-field from the lens feeder. Polarization efficiency $\left(\eta_{pol}\right)$ represents the purity of the radiated E-field polarization. Blocking efficiency $\left(\eta_{block}\right)$, differently to reflector antennas, evaluates the portion of the reflected signal at the lens and propagated back to the feeder. This effect has consequences mainly in the feeder matching coefficient, but it can be disregarded in the total aperture efficiency of the lens antenna. Finally, the surface random error efficiency $\left(\eta_{error}\right)$ estimates the degradation caused by manufacturing defects, such as inadequate flatness of the lens surface. In most of the cases, the polarization, blocking and random errors represent almost negligible contributions to the total aperture efficiency, and the aperture efficiency can be assumed as the total one ($\eta_{Total-aper}\approx \eta_{aper}$).

The total aperture efficiency ($\eta_{Total-aper}$) also relates the antenna directivity $D_{0}$ with the lens aperture area $A_{lens-aper}$ by Equation (2), giving a figure of merit of the amount of lens aperture area that has been used to focus and properly radiate the transmitted E-field from the lens feeder:

(2) $$D_{0}=\frac{4\pi }{{\lambda_{0}}^{2}}\cdot A_{lens-aper}\cdot \eta_{Total-aper}.$$

The realized gain of the antenna, including information of losses and impedance mismatching, is defined as follows:

(3) $$G_{0\_realized}=\eta_{rad}\cdot \left(1-{\left|S_{11}\right|}^{2}\right)\cdot D_{0}=\eta_{rad}\cdot \left(1-{\left|S_{11}\right|}^{2}\right)\cdot \eta_{Total-aper}\cdot \frac{4\pi }{{\lambda_{0}}^{2}}\cdot A_{lens-aper},$$

where $\eta_{rad}$ is defined as the radiation efficiency of the antenna and is giving information about the antenna losses. The antenna-matching coefficient is calculated with the $S_{11}$ scattering parameter. The term $\left(1-{\left|S_{11}\right|}^{2}\right)$ is related to the wasted power due to mismatching effects between the antenna and the transmitter (or receiver) impedances. In the following, the total efficiency can be calculated as:

(4) $$\eta_{tot}=\eta_{rad}\cdot \left(1-{\left|S_{11}\right|}^{2}\right)\cdot \eta_{Total-aper}.$$

### 2.1. Aperture Efficiency Optimization

The amplitude efficiency $\left(\eta_{ampl}\right)$ is dependent on three parameters:

(5) $$\eta_{aper}=\eta_{spill}\cdot \eta_{taper}\cdot \eta_{phase}$$

Spillover efficiency ($\eta_{spill}$) indicates the portion of the radiation signal from the feeder that is not captured and, consequently, not focused by the lens. The taper $\left(\eta_{taper}\right)$ and phase $(\eta_{phase})\;$efficiencies evaluate the uniformity of the amplitude and the phase of the illuminated E-field at the lens aperture. It is not possible to achieve 100% aperture efficiency because always part of the radiated signal by the feeder will not be captured by the lens, which results in worse $\eta_{spill}$. Likewise, the amplitude of the feeder radiation pattern is angle dependent and not uniform. Therefore, the wave will not arrive equally in amplitude at the inner flat surface of the lens. As a result, the $\eta_{taper}$ will be reduced. The phase uniformity of the E-field at the lens aperture, and consequently the $\eta_{phase}$ optimization, will be obtained with an adequate dielectric distribution configuration of the GRIN lens.

#### 2.1.1. Feeder Radiation Pattern Influence

**Figure 1:**
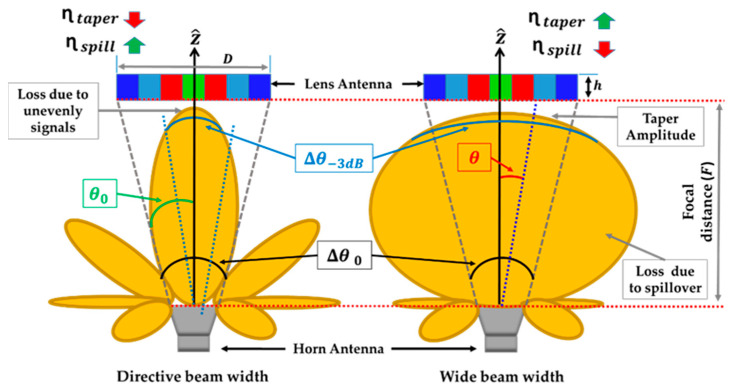
Schematic representation of the relationship between taper efficiency, spillover efficiency, and the beam width of the feed antenna.

Spillover ($\eta_{spill}$) and taper $\left(\eta_{taper}\right)$ efficiencies are inversely related. As seen in Figure 1, two situations are shown where a GRIN lens with several dielectric constant ($\varepsilon_{r}$) rings (represented in distinct colors) is illuminated by two feeders with different beam widths (${∆\theta }_{-3\;dB}$). In both cases, the feeder is located at the same distance from the lens. When the feeder is directive, ${∆\theta }_{-3\;dB}$ is considerably less than $\Delta \theta_{0}$ (angle from the feeder, between the center of the lens antenna and its edges). This makes the width of the radiation pattern of the feeder decrease abruptly from the broadside direction up to the angle $\theta_{0}$ (angle from the feeder between the center of the lens antenna and its edge). As a result, the lens is not evenly illuminated by the feeder.

On one hand, a low value of ${∆\theta }_{-3dB}$ means that most of the power radiated by the feeder is directed toward the lens, and only a small part is wasted in other directions through secondary lobes. Therefore, waste due to spillover is very low, and the $\eta_{spill}$ efficiency is high. On the other hand, the inverse circumstance will occur when the feeder has a large beam width and ${∆\theta }_{-3dB}$ is similar to or even greater than $\theta_{0}$. The lens is illuminated very evenly across the main lobe of the feeder, minimizing taper amplitude and thus maximizing $\eta_{taper}$. However, the broad main lobe causes more power wasted for angles greater than *θ*_0_.

#### 2.1.2. Focal Distance and Lens Diameter

**Figure 2:**
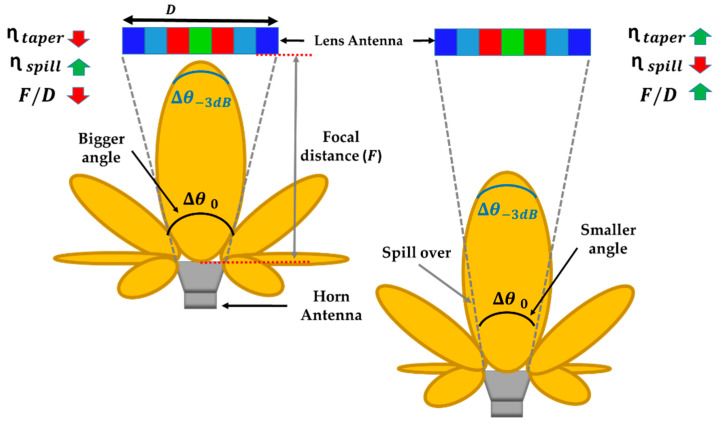
Schematic representation of the relationship between taper efficiency, spillover efficiency, and the F/D ratio.

In addition, $\eta_{spill}$ and $\eta_{taper}$ strongly depend on two parameters: (1) the focal distance (F), which is the distance between the phase center of the feeder and the lens aperture, and (2) the diameter of the lens (D), which determines the F/D ratio. Figure 2 illustrates two scenarios with different F/D ratios in which D is fixed and the feeder to lens separation (F) is varied between a close and a farther distance. As can be seen, increasing F/D implies a reduction of the angle $\theta_{0}$, while in smaller F/D, the angle is wider. As the same feeder is used in both scenarios (same ${∆\theta }_{-3dB}$), the case with higher F/D favors an improvement in $\eta_{taper}$ since the lens is illuminated with a minor arch of the main lobe. However, $\eta_{spill}$ is degraded by wasting more signal that is not captured by the lens. In other situations, the results are opposite. To optimize our design, a balance must be found between $\eta_{spill}$ and $\eta_{taper}$ by controlling the *F* and the D parameters.

### 2.2. Optimum Design

**Figure 3:**
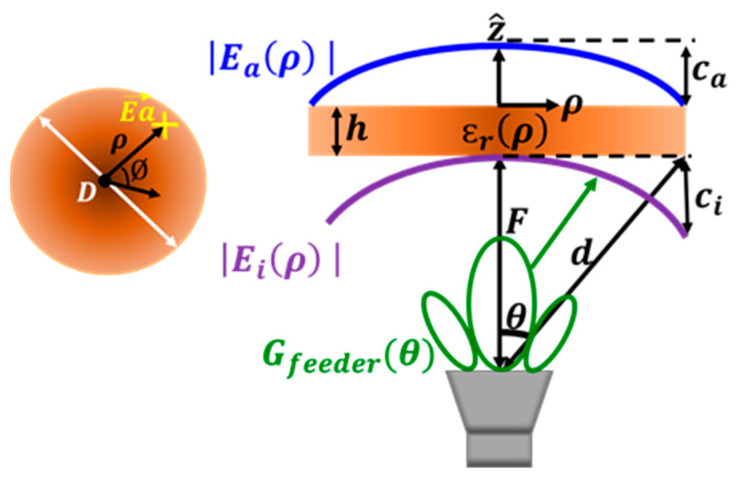
Diagram showing a theoretically based, simplified computation of the electric E-field in the aperture coming from the feed antenna radiation pattern.

Based on the inverse behavior of $\eta_{spill}$ and $\eta_{taper}$, an optimum point can be achieved, which maximizes the amplitude efficiency $\left(\eta_{ampl}\right)$. In that sense, the amplitude of the E-field must be analyzed at the lens aperture ($E_{a}\left(\rho \right)$) following the scheme in Figure 3. This aperture E-field directly depends on the feeder gain pattern $G_{feeder}(\theta )$, which can be analytically characterized by using (6). The index *n* will be defined according to the kind of antenna feeder, and revolution symmetry around $\hat{z}$ axis is considered (feeder radiation patter only depends on θ). For this analysis, the lens has been assumed to have a variably continuous distribution of its dielectric permittivity.

(6) Gfeeder(θ)=2(n+1) cosn(θ), 0≤ θ ≤ π2.

Based on reflectors theory [26], Sec. 15.4.1C, the amplitude of the incident E-field $E_{i}\left(\rho \right)\;$at the inner side of the lens surface can be calculated by (7), where coordinate ρ provides the radial separation between any point on the lens and its center, *d* is the distance between the feeder and the observation point on the lens surface, *P_ent_* is the power delivered to the lens feeder, *η*_0_ is the intrinsic impedance of the medium (air), and *k*_0_ is the wavenumber in the middle (air). As Equation (8) indicates, the ratio between the minimum (achieved at the lens edge, ρ=D/2) and maximum (at the lens center, ρ=0) values of the tapered amplitude representation of the incident E-field in Figure 3 is known as the pedestal ($C_{i}$). As (9) shows, there is a direct relation between the normalized radiation pattern of the lens feeder and the incident E-field pedestal $C_{i}$, including a wave path correction factor related to the edge lens angle $\theta_{0}$.

(7) $$E_{i}\left(\rho \right)=\frac{1}{d(\rho )}\sqrt{\frac{G_{feeder}(\theta )\;P_{ent}\;\eta_{0}}{2\pi }}\;e^{-jk_{0}d(\rho )}.$$

(8) $$C_{i}=\frac{\left|E_{i}\left(\rho =D/2\right)\right|}{\left|E_{i}\left(\rho =0\right)\right|},$$

(9) $$C_{i}\left(dB\right)=10\log\left(\;\frac{G_{feeder}\left(\theta_{0}\right)}{G_{feeder}\left(\theta =0{}^{\circ}\right)}\;\right)+20\;log\left({cos}^{2}(\frac{\theta_{0}}{2})\right).$$

Part of this incident E-field will be reflected when arriving at the lens surface according to the incident reflection coefficient ($\Gamma_{r,i}$), due to the different media at the inner lens interphase. The remaining wave will be propagated inside the lens structure of thickness *h*. It will find different phase constants according to the lens dielectric distribution ($k_{0}\varepsilon_{r}\left(\rho \right))$, up to reaching the outer aperture of the lens, where again there is a change of medium. It is noteworthy that $k_{0}$ presents a phase constant in the air and $\varepsilon_{r}\left(\rho \right)$ stands for the dielectric permittivity at the corresponding radial distance. Therefore, there will be part of the field that will be reflected back ($\Gamma_{r,a}$, reflection coefficient at this point) and the finally transmitted wave configures the E-field amplitude at the outer side of the lens aperture ($E_{a}\left(\rho \right)$), which is computed by (10). For simplicity, the drop amplitude inside the lens propagation due to losses is omitted in this analysis. All the parameters depending on the radial distance ρ are indicated between parenthesis (ρ). Similarly to the incident E-field, in Figure 3 a pedestal value is defined in (11) between the center and the lens edge ($C_{a}$) for the aperture E-field. As will later be detailed, this is a significant design parameter to optimize the lens total aperture efficiency. The distribution of the dielectric permittivity at the GRIN lens originates different reflection values along the lens surface, which explains the differences between both pedestals ($C_{i}\neq C_{a}$).

(10) $$E_{a}\left(\rho \right)=\left|1-\Gamma_{r,a}\left(\rho \right)\right|\left|1-\Gamma_{r,i}\left(\rho \right)\right|E_{i}\left(\rho \right)\;e^{-jk_{0}\varepsilon_{r}\left(\rho \right)h},$$

(11) $$C_{a}=\frac{\left|E_{a}\left(\rho =D/2\right)\right|}{\left|E_{a}\left(\rho =0\right)\right|}.$$

**Figure 4:**
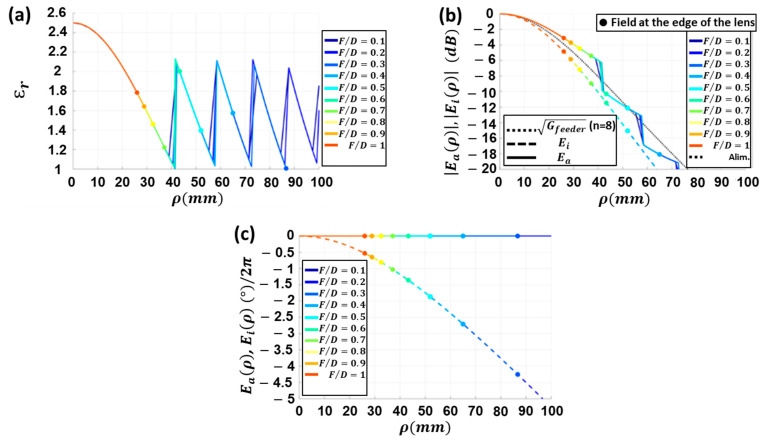
For a lens with $\varepsilon_{r-material}=2.5\;$ and feeder with coefficient n=8 located at F=52 mm: (**a**) radial variation of the permittivity $\varepsilon_{r}$ for each F/D; (**b**) incident field ($\left|E_{i}\left(\rho \right)\right|$) and aperture field ($\left|E_{a}\left(\rho \right)\right|$) at different radial points ρ of the lens for different F/D ratios; (**c**) phase (normalized to 2π) of mentioned fields.

In order to obtain the best possible F/D ratio, $\left|E_{a}\left(\rho \right)\right|$ and $\left|E_{i}\left(\rho \right)\right|$ have been computed in Figure 4 for a feeder with n=8, F=52 mm, $\varepsilon_{r}<=2.5$, and h=25 mm (h is the thickness of the lens antenna) using Equations (7)–(11). 

Figure 4a shows the required distribution of $\varepsilon_{r}$. It decreases towards the edge of the lens antenna to compensate for the largest phase shift at longer propagation paths. When the lens diameter exceeds approximately 40 mm, the value of the $\varepsilon_{r}$ required to reach a uniform E-field phase would be less than 1. Since this is not feasible, a higher $\varepsilon_{r}$ value is sought. The idea is to compensate the phases with a different multiple of 2π radians. This explains the sharp shifts in the curves of $\varepsilon_{r}$ when ρ is, for instance, close to 40 mm.

The normalized amplitude of the aperture E-field for different F/D ratios are given in Figure 4b. As ρ increases, the difference between $\left|E_{i}\left(\rho \right)\right|$ and $G_{feeder}(\rho )$ grows due to the longer propagation path (*d*). Thus, the propagation losses will increase. Due to the smaller diameter *D* and minimal path difference between the center and the edge of the lens, this effect is less noticeable with small lenses (high F/D). Also, it is interesting to note that the distribution of the amplitude of $\left|E_{a}\left(\rho \right)\right|$ has a smoother taper than the incident field $\left|E_{i}\left(\rho \right)\right|$, which is due to a change in the dielectric medium. The distribution of $\varepsilon_{r}$ is a monotonically decreasing curve without discontinuities at the edge for lenses with approximately F/D > 0.7.

The normalized phase distributions of these fields have been calculated in Figure 4c. It can be seen that, as expected, the phase of the incident field deviates more towards the edges of the lens by different propagation distances. It is noted that in the largest lenses, phase differences of several times 2π exist between the center and the edge of the lens. This explains the need to search for $\varepsilon_{r}$ that can equalize phases with differences of multiples of 2π. Likewise, it is evident that the phase of $\;E_{a}\left(\rho \right)$ remains fully equalized thanks to the permittivity distribution imposed on the lens.

**Figure 5:**
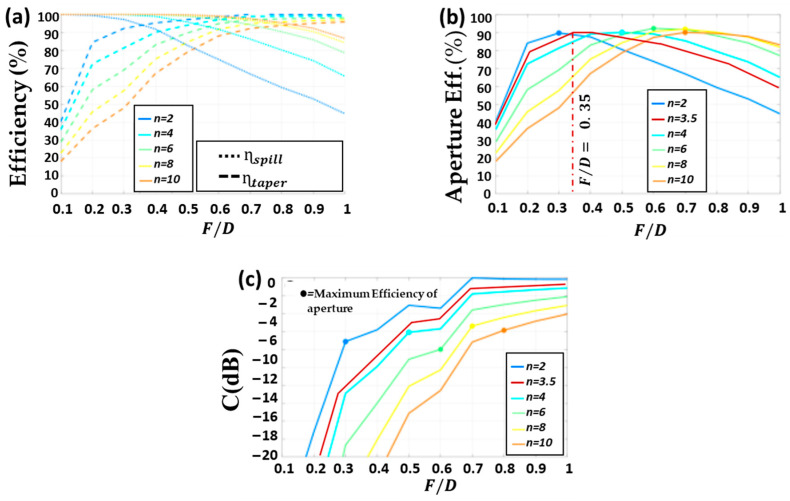
Lens with $\varepsilon_{r-material}=2.5$. (**a**) Taper ($\eta_{taper}$) and spillover efficiency ($\eta_{spill}$) for different feeder diagrams (n) and F/D ratios. (**b**) Aperture efficiency ($\eta_{aper}$ = $\eta_{spill}\cdot$ $\eta_{taper}$) considering uniform aperture E-field phase ($\eta_{phase}=1)$. (**c**) $C_{a}$ of the electric field in the aperture of the lens for different n and F/D.

Depending on the radiation properties of the lens feeder ($G_{feeder}\left(\theta \right)$) and its relative location with the lens (F/D ratio), an optimum lens antenna design can be defined in terms of maximum achievable aperture efficiency ($\eta_{aper}$). Thus, considering an ideal uniform phase distribution of the aperture E-field ($\eta_{phase}=1$), the spillover $\eta_{spill}$ versus the taper $\eta_{aper}$ efficiencies are represented in Figure 5a for several feeders (different n indexes) as a function of the F/D ratio. As in Equations (12)–(14), $\eta_{spill}$, $\eta_{taper}$ and $\eta_{aper}$ directly depend on the gain pattern of the lens feeder ($G_{feeder}\left(\theta \right)$) [26], Sec. 15.4.1C.

(12) $$\eta_{spill}=\frac{\int_{\theta =0}^{\theta_{0}}G_{feeder}\left(\theta \right)sen\theta d\theta }{\int_{\theta =0}^{\pi }G_{feeder}\left(\theta \right)sen\theta d\theta },$$

(13) $$\eta_{taper}=2\frac{{\left|cotan\left(\frac{\theta_{0}}{2}\right)\int_{\theta =0}^{\theta_{0}}\sqrt{G_{feeder}\left(\theta \right)}\cdot tan\left(\frac{\theta }{2}\right)d\theta \right|}^{2}}{\int_{\theta =0}^{\theta_{0}}G_{feeder}\left(\theta \right)sen\theta d\theta },$$

(14) $$\eta_{aper}=\eta_{spill}\cdot \eta_{taper}={\left|cotan\left(\frac{\theta_{0}}{2}\right)\int_{\theta =0}^{\theta_{0}}\sqrt{G_{feeder}\left(\theta \right)}\cdot tan\left(\frac{\theta }{2}\right)d\theta \right|}^{2}.$$

Even when the lens diameter *D* is not very high, a feeder with a small *n* (large beam width) wastes a significant amount of energy by spillover. In contrast, a directional feeder wastes less signal, but has a larger taper amplitude. Nevertheless, for the aperture efficiency ($\eta_{aper}$) representation in Figure 5b an optimum F/D ratio can always be defined for every feeder to obtain the highest efficiency, which in all cases is around 90%. Likewise, Figure 5c represents the pedestal value $C_{a}$ of the E-field amplitude at the lens aperture as a function of the F/D ratio. Consequently, the associated $C_{a}$ pedestal value for an optimum lens design can be calculated with the selected F/D ratio of Figure 5b. As Equation (9) indicates for the incident E-field, there is a direct relation between the pedestal and the feeder radiation pattern. It is computed around 2 dB difference between incident $C_{i}$ and aperture $C_{a}$ pedestals by considering the different reflections at the two media interphases in the lens. Consequently, for an optimum lens design in terms of efficiency, an adequate combination of focal distance (F), lens diameter (D) and feeder radiation pattern is intrinsically required.

### 2.3. Phase Efficiency Optimization

**Figure 6:**
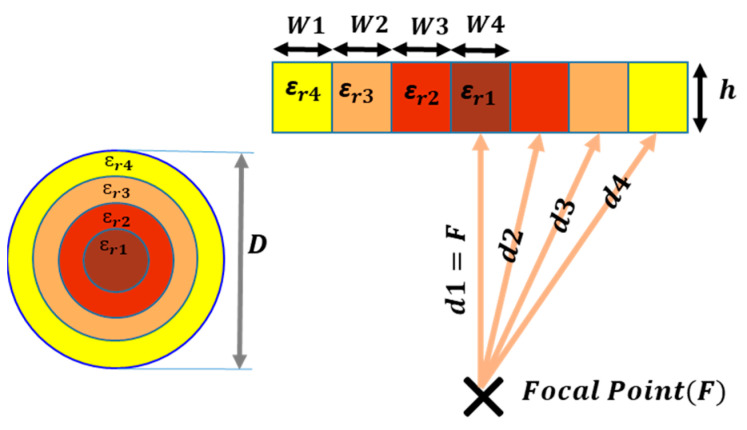
Propagation paths considered for the design of the GRIN lens.

To reach the lens surface, the signals travel different distances. Signals in the center of the feeder travel the shortest distance (F), while those at the edges of the lens travel the longest. The phase difference between signals is caused by the difference in the traveling distances. The phase shift role is an obstacle to achieve 100% phase efficiency, and, as a result, the aperture efficiency will drop. Since the goal is to generate a uniform phase at the lens aperture, the different phase paths should be designed in a way that the propagation paths produce equal phase shift or differ from each other by a multiple of 2π radians. For this reason, as Figure 6 shows, the GRIN lens structure is divided into rings of different dielectric constants. The idea is to achieve equal propagated E-field phase at the lens aperture according to (15):

(15) $$k_{0}d_{1}+k_{0}\sqrt{\varepsilon_{r1}}h=k_{0}d_{2}+k_{0}\sqrt{\varepsilon_{r2}}h=k_{0}d_{3}+k_{0}\sqrt{\varepsilon_{r3}}h=k_{0}d_{4}+k_{0}\sqrt{\varepsilon_{r4}}h.$$

In this way, the electrical path will be shortened without effecting the phase uniformity at the aperture. It should be noted that the equality in the phase is only possible at one frequency. In order to reach a uniform phase, the lens must have continuous permittivity distribution. Therefore, GRIN lens can be a good candidate to obtain the desired results. In addition, to select the appropriate lens thickness (h), it is required that

(16) $$\varepsilon_{ri}={\left(\frac{k_{0}F+k_{0}\sqrt{\varepsilon_{r1}}h-k_{0}d_{i}+n2\pi }{k_{0}h}\right)}^{2}.$$

The proposed $\varepsilon_{ri}$ depends on the focal distance F, and the thickness h. As it is mentioned before, a phase difference of 2π can be allowed to exist between different paths ($d_{1}-d_{4}$) without degrading the phase uniformity (see Figure 6).

### 2.4. Perforated GRIN Lens

**Figure 7:**
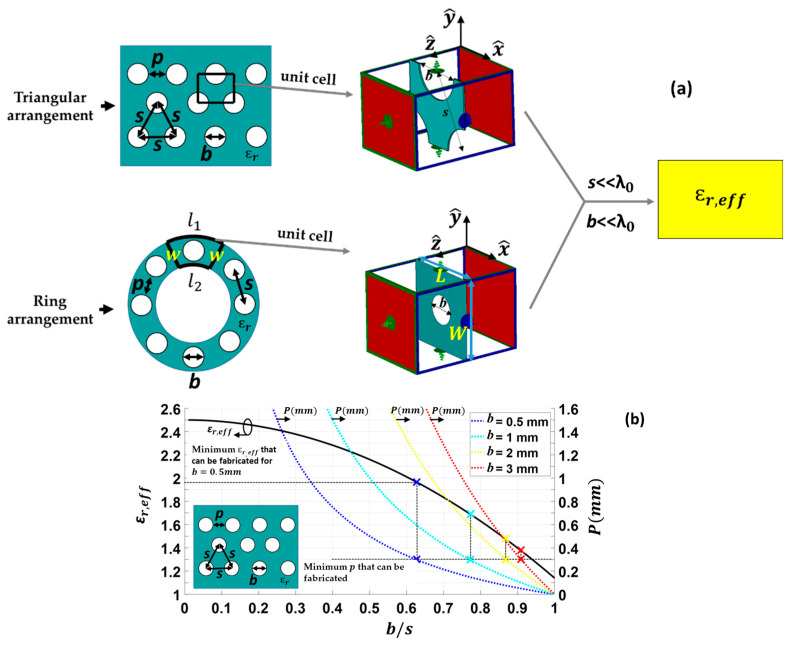
The fabrication limitation. (**a**) Different arrangement of the holes: triangular arrangement and ring arrangement; (**b**) $\varepsilon_{r,eff}$ for different value of b/s in a triangular arrangement for a material with $\varepsilon_{r}=2.5$.

The radial pattern of the dielectric permittivity in a GRIN lens can be synthesized by including several perforated rings with different widths and normally equal heights. In this way, when the lens is illuminated by the spherical wave of an antenna feed, a plane wave with a uniform phase is generated. GRIN lenses are popular for their easy manufacturing (use of a single material) and flexibility. The base material is a dielectric base with nominal dielectric constant ɛ*_r_*. A common method of synthesizing the different required values of the *i*-th ring permittivities ($\varepsilon_{ri}$ in Figure 6) in the lens is by making perforations. As the number of perforations in a lens ring increases, its equivalent effective permittivity $\varepsilon_{r,eff}$ decreases. Therefore, a base material with high nominal permittivity $\varepsilon_{r}$ provides a wider range of possible values of dielectric constants, allowing more flexibility in the lens design. The effective permittivity of a perforated dielectric can be calculated according to (17) and (18):

(17) $$\varepsilon_{r,eff}=\varepsilon_{r}(1-\alpha )+\alpha ,$$

(18) $$\alpha =\frac{Area\;of\;the\;holes}{Total\;area},$$

where α depends on the physical arrangement of the holes. A triangular ordering has been commonly used, as in [27], to place the holes. In this paper, a concentric ring disposition has been chosen (Figure 7). This reduces each ring width and enables a wider range of permittivities with more design freedom.

According to Figure 7a, b, p and s represent the diameter of the holes, thickness of solid dielectric walls, and the distance between the centers of two adjacent holes, respectively. It is noteworthy that parameters b and s are much lower than the wavelength ($b\ll \lambda_{0}$ and $s\ll \lambda_{0}$). These parameters determine the $\varepsilon_{r,eff}$ that will be explained in the following. Several simulations have been performed in Figure 7b for a unit cell in triangle arrangement with different b/s ratios (between 0.1 and 1). When the b/s ratio is high to obtain low values of $\varepsilon_{r,eff}$, the density of holes in the same area grows. Thus, the thickness of the walls (p) that lies between holes decreases. Each curve in Figure 7b corresponds to a distinct value of b, and numerous p values are determined for various values of b/s. The graph shows that higher diameters of b allow higher values of p for a given value of $\varepsilon_{r,eff}$. This guarantees the mechanical stability of the structure. Therefore, it is convenient in the design to reach a compromise where b is as small as physically possible.

In this work, all prototypes have been manufactured by 3D printing using stereolithography. The base material is a resin with dielectric permittivity $\varepsilon_{r}=2.5$ and a minimum manufacturable hole diameter of 1 mm. Another limitation for fabrication is the thickness of the wall (p). If p is too low, the hole will become very brittle and will break easily. To maximize the range of feasible $\varepsilon_{r,eff},\;$it was determined that p<0.3 mm led to breakage and brittleness in the fabricated prototypes. For instance, according to Figure 7b, if a hole diameter b=2 mm is taken, then the minimum achievable value of effective permittivity for triangular arrangement is $\varepsilon_{r,eff,min}=1.48$. These restrictions should be considered during the design process.

Each air hole with its surrounding is considered as a unit cell with W×L dimensions. W is the approximation of the ring widths (parameter of w in Figure 7a) and L is the planar approximation of the angular arc distance between two adjacent holes in the same ring (parameters of $l_{1}$ and $l_{2}$ in Figure 7a). According to this, the $\varepsilon_{r,eff}$ of each ring can be computed with Equations (17) and (18). There is no requirement for the ring widths (W) to be equal. When compared to triangular arrangements, this can be advantageous since it allows for an increase in the number of rings. It also allows for the discriminating of various dielectric permittivity values.

A unit cell is simulated to achieve the permittivity. Taking advantage of the symmetric arrangement of the holes in each ring, by using unit cell modeling, there is no need to simulate the whole ring to obtain the equivalent $\varepsilon_{r}$. This saves computing time for each simulation. The unit cell is in fact a trapezoid, but given the difficulty of representing this type of geometry in both theory and CST Microwave Studio, a rectangle will be defined, assuming some errors. For example, for a small quadrangular sheet (11 mm×11 mm) of dielectric constant $\varepsilon_{r}=2.5$ (resin) and thickness h=0.005 mm with 4 holes (r=2 mm) in the sheet, the simulation provides $\varepsilon_{r,eff}=1.8726$. The attained $\varepsilon_{r,eff}$ by using Equations (17) and (18) is 1.8769, which is close to the simulated one. Although the positions of the holes are not crucial, the size of the holes, the number of the holes and the total size of the unit cell determine the ${\varepsilon_{r}}_{eff}$. In addition, making a realistic prototype of the GRIN lens is not possible without accounting for the restrictions of the manufacturing process. In this project, a 3D printer of the Formlabs brand (stereolithography process) is used. In this design, the b (diameter) value is 2 mm to avoid any fabrication problem.

## 3. Linearly Polarized Flat Lens Antenna Fed by a Pyramidal Horn Antenna (LP-FLA + PHA)

### 3.1. Design Description

**Figure 8:**
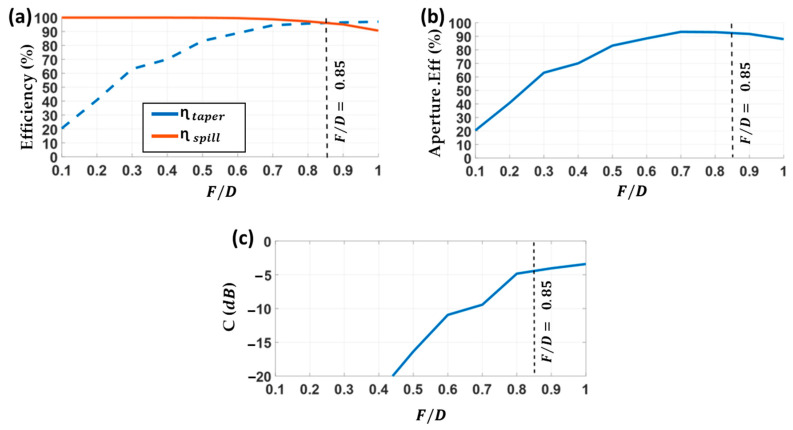
Analyzing the effect of changing the F⁄D ratio: (**a**) taper efficiency ($\eta_{taper}$) and spillover efficiency ($\eta_{spill}$), (**b**) aperture efficiency ($\eta_{aper}$*=*$\eta_{spill}\cdot$ $\eta_{taper}$) considering uniform aperture E-field phase ($\eta_{phase}=1)$, (**c**) amplitude pedestal $C_{a}$ of the E-field at the lens aperture for antenna feeder with index n=9.5.

In order to verify the optimum design process detailed in Section 2, a Linearly Polarized Flat GRIN Lens Antenna (LP-FLA) based on a concentric hole ring arrangement has been designed. It has a pyramidal horn with a rectangular aperture as an antenna feeder radiating a linear vertical polarization. This antenna is suitable for terrestrial high data rate and low latency 5G communications at the 28 GHz band. The pyramidal horn antenna does not have a symmetrical radiation pattern at the H- and E-planes. A suitable n=9.5 index in the H-plane and n=8.3 in the E-plane have been estimated for the theoretical antenna feeder characterization of (6) by using CST Microwave Studio simulations. Then, the $\eta_{spill}$ and $\eta_{taper}$ curves of Figure 8a for different F/D ratios have been computed by using (12) and (13), respectively.

When the horn feeder (PHA) is moved away from the lens antenna (LP-FLA), $\eta_{taper}$ increases significantly, while $\eta_{spill}$ decreases with small changes. The combination of both in (14) provides the aperture efficiency $\eta_{aper}$ representation of Figure 8b, in which the phase efficiency is supposedly ideal ($\eta_{phase}=1$). The results indicate that when selecting F/D=0.85, a maximum $\eta_{aper}$ value around 90% is expected for the LP-FLA + PHA system. The corresponding pedestal of the lens aperture E-field amplitude is $C_{a}\approx -5\;dB$ for the H-plane. Therefore, D=75 mm and F=64 mm are considered for the lens diameter and focal distance, respectively.

The geometrical F and D lens parameters have been determined for the optimum design of the E-field amplitude at the aperture ($\left|E_{a}\left(\rho \right)\right|)$. Now, the GRIN dielectric permittivity distribution in the rings must be designed to achieve the uniformity of the E-field phase at the lens aperture, considering the $\eta_{phase}=1$ premise. As was explained in Section 2.4, the GRIN lens is implemented with hole perforations to obtain the different dielectric permittivity values. This hole arrangement enables us to increase the number of rings compared to other hole dispositions (i.e., [27]), allowing higher permittivity resolution along the lens surface.

**Figure 9:**
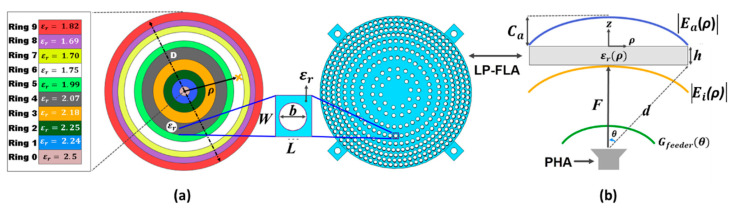
LP-FLA. (**a**) The GRIN lens antenna with 10 different $\varepsilon_{r,eff}$. (**b**) The electric fields from the feeder to the aperture of the lens antenna.

In Figure 9a, 10 rings (named from i=0 to 9) with 10 different dielectric constants $\varepsilon_{ri}$ have been considered. They will later be implemented with 10 perforated concentric hole rings (Figure 9b) following the unit cell characterization explained in Section 2.4. The central ring presents the permittivity of the non-perforated base dielectric material ($\varepsilon_{r0}=2.5$). The lower defined dielectric constant $\varepsilon_{r8}=1.69$ (i=8 ring) is related with the minimum realizable hole diameter in the lens fabrication. In fact, the lowest attainable effective dielectric permittivity with this ring arrangement, due to manufacturing limitations, is 1.69. This ring configuration, with its associated effective dielectric permittivities, has been designed to achieve E-field uniform phase at the lens aperture (z=0, in Figure 9b) as detailed in Section 2.3.

In order to check the design correspondence with the optimum case, the amplitude of the incident ($\left|E_{i}\left(\rho \right)\right|$) and aperture ($\left|E_{a}\left(\rho \right)\right|$) E-fields have been calculated by using (7) and (10), respectively. For (7), the ideal continuous variation of the lens dielectric constant $\varepsilon_{r}\left(\rho \right)$ is sampled into 10 values ($\varepsilon_{r0}$ to $\varepsilon_{r9}$) applied to the 10 annular ring regions shown in Figure 9. For an appropriate unit cell definition for the perforated equivalent ring in Figure 9a, the length (arc, L) parameter has been chosen by dividing each ring into equal parts taking into account the number of holes for the ring.

The characteristic parameters of the rings are summarized in Table 1: the interior radial distances (from the lens center) where the rings star ($\rho_{int}$), the external radial distances where the rings finish ($\rho_{ext}$), the number of holes, and, the simulated effective dielectric constants ($\varepsilon_{ri,eff}$). They practically match the theoretical values of Figure 9a. They decrease from the center of the first ring to the eighth ring, while increase from ring 8 to ring 9 (1.69 to 1.82). This change is due to the fabrication limitations of b and p parameters (Figure 7). A higher number of holes would have been required for ring 9 to fulfill the uniform phase condition, but the associated wall thickness of the ring (p) would have been un-fabricable. Therefore, in this ring the phase has been equalized with 2π radians difference as in Figure 4a was shown. It should be noted that the equivalent effective permittivity in every ring ($\varepsilon_{ri,eff}$) is implemented with a different air-filled hole density. Thus, their associated losses for the wave propagation inside the structure are not the same. This aspect will modify the amplitude of the E-field at the lens aperture ($\left|E_{a}\left(\rho \right)\right|$).

**Figure 10:**
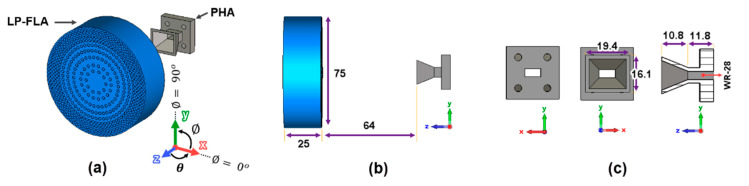
Schematic of the pyramidal horn antenna and the flat lens. (**a**) Lens antenna in front of PHA. (**b**) Overall dimensions of the system. (**c**) Dimensions of the pyramidal horn antenna (dimensions in mm).

According to (7) and (10), the gain pattern of the lens feeder $G_{feeder}\left(\theta \right)$ is required to represent $\left|E_{i}\left(\rho \right)\right|$ and $\left|E_{a}\left(\rho \right)\right|$. In this case, the simulation with CST Microwave Studio has been used for the radiation pattern coming from the pyramidal horn antenna (PHA), instead of the theoretical approximation of (6) with the index n=9.5. Figure 10a illustrates the complete linearly polarized flat 3D dielectric GRIN lens antenna (LP-FLA) in front of the pyramidal horn antenna (PHA) feeder. The thickness of the perforated GRIN lens is fixed to h=25 mm (Figure 10b) and the dimensions of the PHA are detailed in Figure 10c. A standard WR-28 [28] waveguide section has been used to feed the PHA with the ${TE}_{10}$ fundamental mode, vertically polarized along the $\hat{y}$ direction.

**Figure 11:**
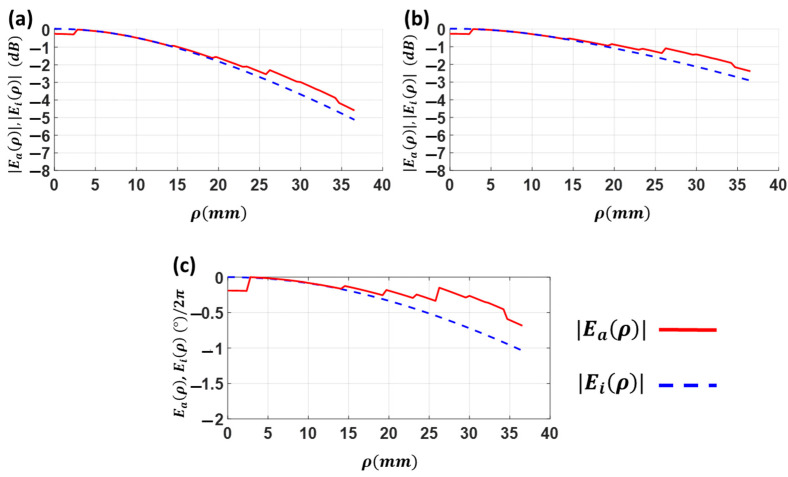
Analyzing the field. (**a**) Amplitude of the incident electrical field $\left|E_{i}\left(\rho \right)\right|$ and the electrical field at the aperture $\left|E_{a}\left(\rho \right)\right|$ at different radial points ρ of the LP-FLA in XZ-plane. (**b**) Amplitude of the incident electrical field $\left|E_{i}\left(\rho \right)\right|$ and the electrical field at the aperture $\left|E_{a}\left(\rho \right)\right|$ at different radial points ρ of the LP-FLA in YZ-plane. (**c**) Phase of the incident electrical field $E_{i}\left(\rho \right)$ and the electrical field at the aperture $E_{a}\left(\rho \right)$ at different radial points ρ of the LP-FLA in YZ-plane.

The normalized results of the amplitude and phase of the incident E-field ($E_{i}\left(\rho \right)$, interface at z=−h in Figure 9b) and the aperture E-field ($E_{a}\left(\rho \right)$, interface at z=0) are shown in Figure 11a–c, at different ρ values. The obtained pedestals at the lens edge (ρ=37.5 mm) for H-plane are $C_{i}\approx -5\;dB$ for the incident E-field and $C_{a}\approx -4.5\;dB$ for the aperture E-field. The pedestals for the E-plane are $C_{i}\approx -3\;dB$ for the incident E-field and $C_{a}\approx -2\;dB$ for the aperture E-field. This means around 1 dB error compared to the theoretical optimum value ($C_{a}\approx -5\;dB$) in the H-plane, mainly due to the different losses in between the hole rings.

Making more holes reduces the permittivity of the dielectric. This also increases the air portion and consequently reduces the ring losses. In fact, the highest losses are found at the lens center where no holes are included. The lowest losses are found at the edge ring with the largest number of air-filled holes. These losses were not considered in the theoretical model of Section 2. In the phase case, Figure 11 demonstrates that the phase uniformity is better approximated at the aperture compared to the incident lens face, with a peak phase difference of 0.5 radians (28°). It should be noted that due to the lens symmetry, the phase of the E-fields in both the E-plane and the H-plane is the same.

### 3.2. Experimental Results

**Figure 12:**
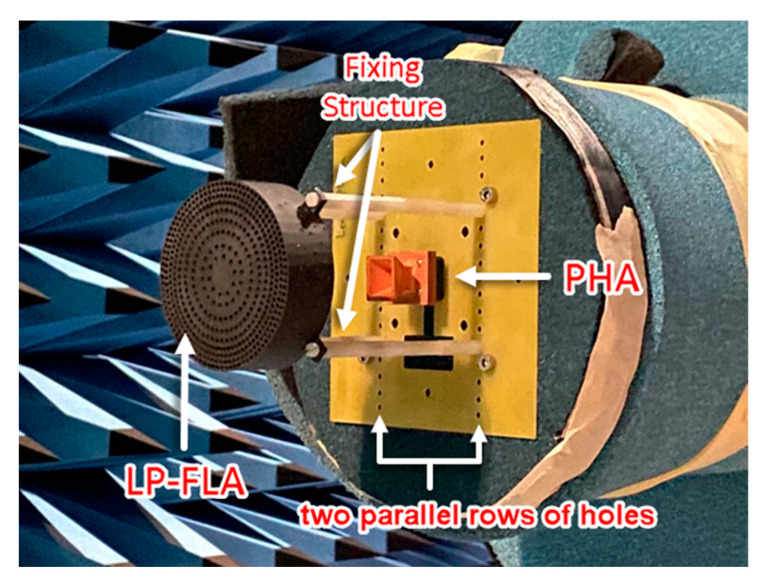
Manufactured prototype of the LP-FLA + PHA system.

A prototype of the LP-FLA fed by a PHA (Figure 12) has been manufactured by 3D printing based on stereolithography. It has been complemented with a copper coating process to metalize the surface of the PHA. The prototype has been measured and compared with simulations. A very wide frequency band response is achieved in terms of matching for the PHA feeder measured alone. The frequency band defined for the systems spans from 26 to 30 GHz (14% fractional bandwidth). The PHA realized gain is over 11.5 dBi in this frequency band, with a 0.3 dB reduction compared to the directivity associated with losses of the copper coating process. Figure 12 also shows the nylon fixing supports that were used to place the LP-FLA in front of the PHA at the focal distance *F*.

**Figure 13:**
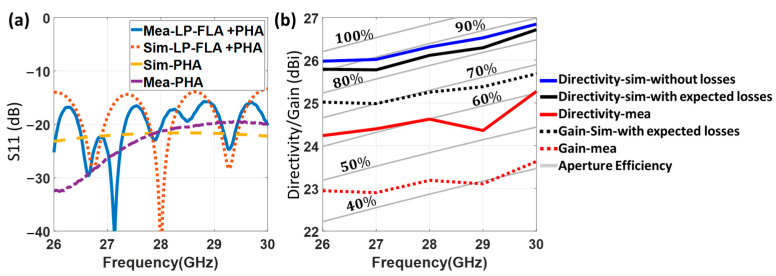
Measured and simulated results. (**a**) Reflection coefficient for the LP-FLA + PHA. (**b**) Aperture efficiency, realized gain and directivity of the proposed LP-FLA + PHA.

The measured and simulated results of the full prototype (LP-FLA + PHA) for the reflection coefficient are shown in Figure 13a. A good concordance is observed between simulation and measurement, with a response below −15 dB in the whole frequency band. The PHA feeder response without the lens in front is also included in the figure to evaluate the mismatching that the reflected wave at the lens surface generates over the PHA. In this case, the PHA reflection coefficient is under −20 dB. It is obvious that including the lens antenna in front of the PHA affects the reflection coefficient of the antenna system.

The measured and simulated peak values of directivity and realized gain for the LP-FLA + PHA prototype are illustrated in Figure 13b. The corresponding total aperture efficiency lines are also added to the figure (gray lines). The LP-FLA is intended to operate at its maximum total aperture efficiency (around 90% according to Figure 8a), which is greater than 80% in simulations. Two perspectives on the simulated directivity are considered. One includes the specified datasheet losses of the substrate (resin) material implementing the lens (loss tangent =0.018). The second does not include losses.

The highest simulated directivity in the case without loss is 26.85 dBi at 30 GHz, with an average total aperture efficiency around the theoretical 90% value. This directivity unexpectedly drops to 26.7 dBi when the resin loss tangent is considered in the simulation. This effect is a very particular aspect of perforated GRIN lens antennas that will be explained in the following. Nevertheless, the obtained values of the simulated total aperture efficiency and directivity, significantly improve conventional designs: 38% average aperture efficiency and directivity between 21.6 and 22.9 dBi at the same frequency range and with the same lens size as [27]. The lens and feeder interaction in [27] was not conveniently modeled for obtaining an optimum performance, which in turns was a goal of the present work. The measured directivity is over 24 dBi in all the frequency band with a peak value of 25.3 dBi at 30 GHz and a 60% average total aperture efficiency.

As previously stated, losses in the lens substrate are not usually considered during the design procedure described in Section 2. However, in contrast to some conventional antennas, here losses have a remarkable impact on the directivity, as seen by the simulated directivity with and without losses. The reason is that the wave transmitted inside the lens up to its aperture travels through the 10 rings. They have varying losses due to the variable air density of their perforations. As a result, the amplitude of the aperture E-field ($\left|E_{a}\left(\rho \right)\right|$) changes in comparison to the no-losses case. Therefore, the pedestal ($C_{a}$) is different from the ideal case assumed in its design, and the total aperture efficiency is now lower.

**Figure 14:**
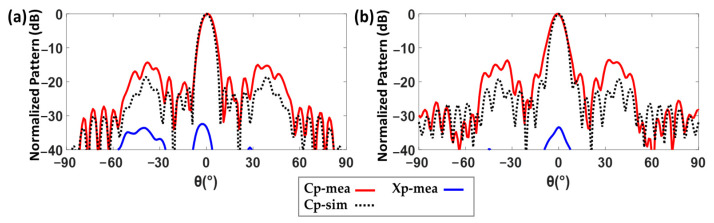

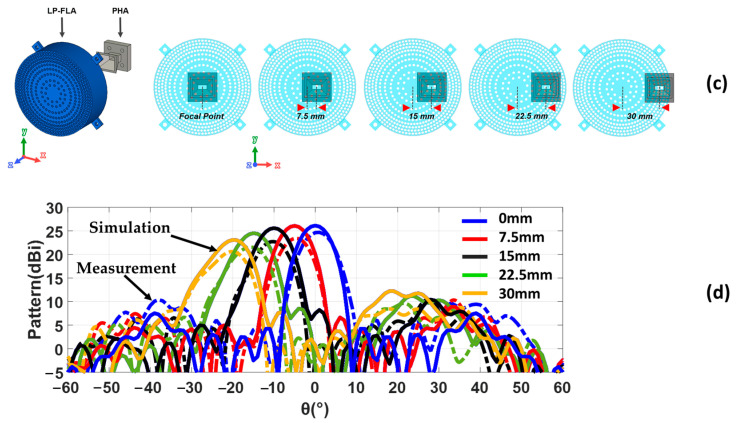
(**a**) Normalized radiation pattern—ϕ=0°, H plane (simulation and measurement). (**b**) Normalized radiation pattern—ϕ=90°, E-plane (simulation and measurement). (**c**) Different x offset positions of the feeder from the centered lens focal point. (**d**) Measured and simulated radiation pattern (directivity) of the structure at different positions (ϕ=90°).

A drop of the measured realized gain of approximately 1.4 dB is observed in comparison with the measured directivity. It is exclusively due to the reduction of the radiated power associated with the loss tangent of the lens substrate. Therefore, this difference is not related to the pedestal ($C_{a}$) modification of the aperture E-field amplitude, which degraded the directivity with respect to the ideal simulation. In more detail, as in the PHA realized gain measurement was indicated, 0.3 dB of the 1.4  dB realized gain reduction comes from the lens feeder and is related to the PHA copper coating process. As the feeder is not symmetric and the aperture is rectangular, the measured and simulated normalized radiation pattern at the central design frequency (28 GHz) in the H-plane and E-plane are compared in Figure 14a,b. A increase in the side lobes (SLL) is observed in both planes, as well as a main beam widening at the E-plane. In addition, a null filling in between the main and the two lateral side lobes is detected in both planes. These effects can explain the differences between the measured and simulated directivity (with substrate losses). On the other hand, a very good polarization antenna performance is achieved, with a cross polar level below −30 dB. 

These kinds of lens antennas are planned to be part of a MIMO system for 5G applications. Thus, the directivity pattern at the antenna H-plane (XZ, ϕ=0°, in Figure 14) has also been measured at 28 GHz for different x-offsets of the feeder from the centered focal point positions of the lens, according to the scheme in Figure 14c. Later, measurements are compared with simulations (with losses) in Figure 14d. The two columns of aligned extra holes located in the fiberglass plate of the measurement system in Figure 12 allow displacing the lens location of the PHA feeding position. Exciting the lens from the lens focal point (x=0 mm offset) leads to a broad side pattern and lower side lobe level. When the feeder moves away from the lens focal point, the scanning capability of the main beam is achieved, but the directivity decreases and the side lobe level increases. The feeder offset displacement provides a shift in the aperture E-field phase that directly leads to a drop in the total aperture efficiency and directivity. From the maximum measured value of 24.7 dBi for broadside excitation (x=0 mm), the directivity decreases to 20.6 dBi for a steering angle of 20° (x=30 mm*)*. A coverage of 28° with directivity higher than 15 dBi is attained. As the lens is symmetric, several feeders can be placed to have independent beams and reach a MIMO high-directive symmetric 2D coverage from −20° to 20°.

## 4. Circularly Polarized Stepped Lens Antenna Fed by a Bow-Tie Cavity Waveguide Antenna (CP-SLA + BCA) for Satellite 5G Applications

### 4.1. Design Description

In this section, a novel concept of a Circularly Polarized Stepped Lens Antenna (CP-SLA) fed by a Bow-tie Cavity Antenna (BCA) is introduced. The proposed system is designed for 5G communications and satellite applications, operating between 26 GHz and 30 GHz. The design of the CP-SLA involved two challenges. The first challenge was to increase the total aperture efficiency by improving the phase uniformity at the aperture of the lens. The second goal was to reduce the focal distance by placing the feeder closer to the lens antenna, leading to a more compact system. For this purpose, a horn feeder could not be placed close to the lens antenna because of its high directivity, which would lead to high reflection. Therefore, a unique, less directive, circular polarized bow-tie cavity antenna (BCA) is introduced, instead of the horn that was used in previous section.

**Figure 15:**
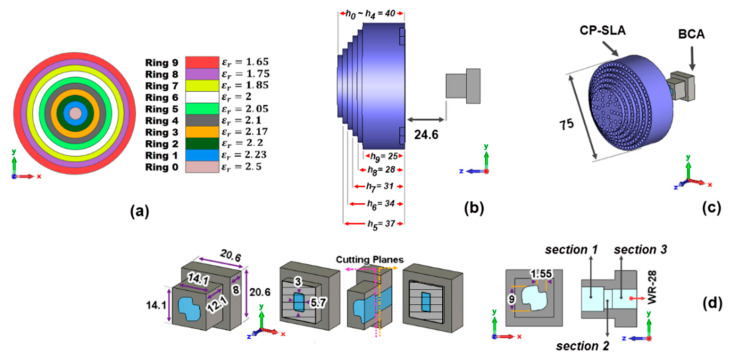
Schematic of the bow-tie antenna and the stepped lens. (**a**) Stepped lens permittivities. (**b**) Thickness of the CP-SLA. (**c**) CP-SLA + BCA. (**d**) Dimensions (mm) of the bow-tie cavity.

The permittivities related to the CP-SLA are presented in Figure 15a. The number of holes increases from ring 1 to ring 9. This provides different $\varepsilon_{r,eff}$, which are computed by the unit cell approach explained in Section 2. The $\varepsilon_{r,eff}$, starting from the center of the lens antenna, decreases from 2.5 to 1.65. Table 2 summarizes the dimensions, permittivity values $\varepsilon_{ri,eff}$, the number of holes, and the heights of the 10 rings compounding the stepped lens in Figure 15a. The LP-FLA had rings with unequal widths. Unlike the LP-FLA, the width of each ring in the CP-SLA is close to the width of the neighboring rings.

The smallest $\varepsilon_{r,eff}$ in the last ring 9 in Figure 9a, as previously mentioned, could not be achieved in the LP-FLA design. This required the use of several holes, which made the fabrication of the ring almost impossible. Consequently, 2π radians shift (see Figure 4a) was used to address this issue, and the $\varepsilon_{r,eff}$ in this ring was higher than in the preceding ring (ring 8, Figure 9a). This problem has been solved in the CP-SLA. By employing varied thicknesses in the design of the rings, it is feasible to synthesize the minimum $\varepsilon_{r,eff}$ in the final ring, avoiding the 2π radians turn. Moreover, the phase dispersion in frequency is higher when a 2π radians shift is implemented, because at the central frequency the phase is the same for all the rings. Out of the design frequency, the phase changes more quickly in the path having 2π radians shift in comparison to the others. Thus, for avoiding the 2π radians shift compensation, the paths are modified physically to equalize the phase more easily.

The design now includes 10 rings with different thicknesses as depicted in Figure 15b. It starts from a circle without perforated areas, which is located at the center of the lens (ring 0) with radius r=3.75 mm. Up to ring 4 with radius r=18.75 mm, the thickness of the rings is the same and equal to 40 mm. In the following, from ring 5, the thickness of the rings gradually decreases to the last value of 25 mm. To have an equal propagated E-field phase at the lens aperture, Equation (19) has been employed in the designing process:

(19) $$k_{0}d_{1}+k_{0}\sqrt{\varepsilon_{r1}}h_{0}=k_{0}d_{2}+k_{0}\sqrt{\varepsilon_{r2}}h_{1}=k_{0}d_{3}+k_{0}\sqrt{\varepsilon_{r3}}h_{2}=k_{0}d_{4}+k_{0}\sqrt{\varepsilon_{r4}}h_{3}.$$

In this design, the diameter of the CP-SLA is the same as for the LP-FLA (D=75 mm). The focal distance (F) is significantly smaller: 24.6 mm in the stepped lens versus 64 mm in the flat lens. It is presented in Figure 15b,c. Less volume in the entire system is, of course, a benefit of the reduced focal distance. However, special attention should be given to the challenges that occur because of the changes in the radiation pattern of the feeder. According to Section 3.1, the n value of the BCA in (6) is 3.5. This provides a wider radiation pattern than the PHA, allowing a smaller F for feeding the CP-SLA. On the other hand, because this system is intended for 5G satellite applications, circular polarization is required. A horn antenna could be also designed for circular polarization. However, with such a close focal distance, horn antennas cannot provide an adequate radiation pattern. It is also noteworthy that, in many cases, GRIN lens antennas are fed directly by open rectangular waveguides, without a horn. Thus, the designer does not control the radiation pattern of these open-ended rectangular waveguides, as is performed with the BCA in the proposed design.

Additional features of the BCA are introduced in the following. A low-cost, circularly polarized, 3D printed feeder is pursued, as illustrated in Figure 15d. The concept of this antenna was firstly introduced by some of the authors in [24]. It is used to radiate a circularly polarized signal by transforming the linearly polarized E-field of the main mode propagated inside a rectangular waveguide. It is an aperture type antenna where the radiating aperture has a bow-tie shape, hence its name. Three guiding sections made up the antenna (see Figure 15d). Section one corresponds to the bow-tie-shaped radiating cavity. Section two serves as an adapter between section one and section three. Lastly, section three is a standard WR-28 rectangular waveguide. The operation of this antenna consists of transforming the field of the fundamental mode ${TE}_{10}$ of the rectangular waveguide, linearly polarized, until obtaining at the output of the radiating cavity a field with Right (RHCP) or Left (*LHCP*) Handed Circular Polarization, depending on the bow-tie inclination sense. When a square waveguide replaces the rectangular one, dual circular polarization can be obtained. Each of the two fundamental modes propagated in the square waveguide (${TE}_{10}$ linearly polarized according to $\hat{y}$, ${TE}_{01}$ linearly polarized according to $\hat{x}$), generates circular signals with opposite rotation directions. Thus, ${TE}_{10}$ gives rise to RHCP and ${TE}_{01}$ to LHCP radiation.

### 4.2. Experimental Results

**Figure 16:**
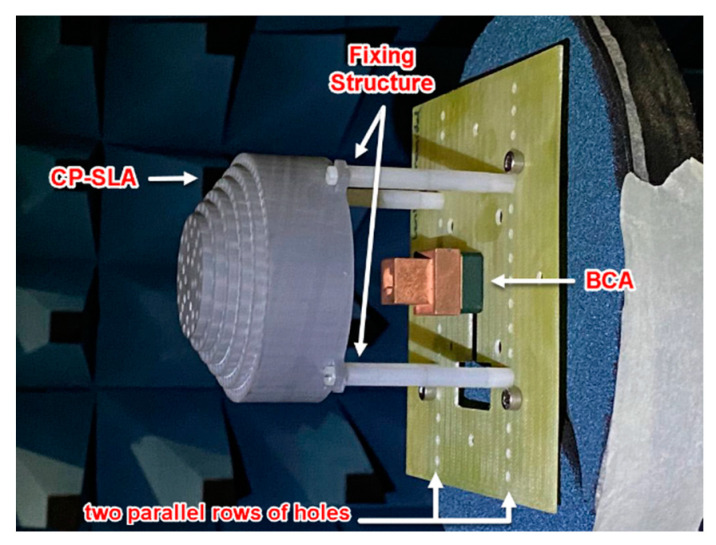
Manufactured prototype of the CP-SLA + BCA system.

**Figure 17:**
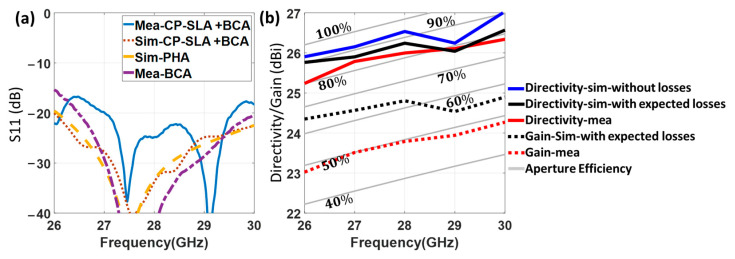
Measured and simulated results. (**a**) Reflection coefficient of CP-SLA + BCA. (**b**) Total aperture efficiency, realized gain and directivity of CP-SLA + BCA.

As in the case of the flat lens antenna, a prototype of the CP-SLA fed by a BCA (Figure 16) has also been manufactured by the 3D printing process based on stereolithography. The prototype has been measured and compared to simulations for evaluating its performance. The reflection coefficient of this structure is illustrated in Figure 17a. It is below −15 dB in both experimental and simulation results in its entire frequency range. To assess the mismatching that the reflected wave at the lens surface causes, the single BCA feeder response without the lens in front is also presented. The simulated reflection coefficients for both the CP-SLA + BCA and the isolated BCA are below −20 dB, with a quite similar response. This demonstrates that, although the focal distance is significantly shorter in this design, there is lower mutual interaction between the lens and the feeder in comparison to the FLA + PHA prototype. The BCA directivity reduction is more significant than the closer lens approximation. Nevertheless, slight differences between measured and theoretical results are shown. Overall, the reflection performance of the whole structure is appropriate.

As in the LP-FLA + PHA case, directivity and realized gain (with and without considering substrate losses in the lens) have been measured and compared with the simulated results in Figure 17b. The figure includes the relevant total aperture efficiency lines (gray lines). The stepped lens antenna is intended to operate at its maximum total aperture efficiency, which in simulation is around 90%. Two viewpoints on the simulated directivity have been considered, as for the LP-FLA + PHA. One is with expected loss (loss tangent =0.018). The other is without loss.

It is found that the losses effect in the CP-SLA + BCA is higher than in the LP-FLA + PHA system. Accordingly, the peak directivities at 30 GHz are simulated as 26.6 dBi with loss and 27 dBi without loss for the CP-SLA + BCA system. For the LP-FLA + PHA system at the same frequency, these values were 26.7 dBi with loss and 26.8 dBi without loss (see Figure 13b). The loss impact on the CP-SLA + BCA directivity is greater than in the LP-FLA + PHA case. However, the measured directivity result (red line) in the desired frequency band ranges from 25.2 dBi to 26.3 dBi, which is very close to the simulation results. In fact, the agreement between simulation and measured results can be explained by an improved phase stability at the aperture of the stepped lens. Additionally, the measured realized gain (dotted red line) changes from 23 dBi to 24.3 dBi in the desired frequency band. In this case, a gain drop of 2.2 dBi is observed when compared to the measured directivity (red line). It can be concluded that the stepped lens antenna contains numerous rings with greater thickness, resulting in a higher gain drop compared to the flat lens antenna (1.4 dBi in Figure 13b).

In conclusion, although the CP-SLA configuration is expected to end up resulting in higher losses, it has superior performance in terms of the phase uniformity at the aperture. This influences the pedestal ($C_{a}$) and makes the measured directivity almost identical to the simulated one. It thus offers a unique advantage. In this situation, the realistic total aperture efficiency validates the superior performance of the structure. Notably, as the BCA feeder provides Right Handed Circular Polarization (RHCP), the CP-SLA + BCA has symmetric radiation patterns in both main planes, unlike the LP-FLA + PHA.

**Figure 18:**
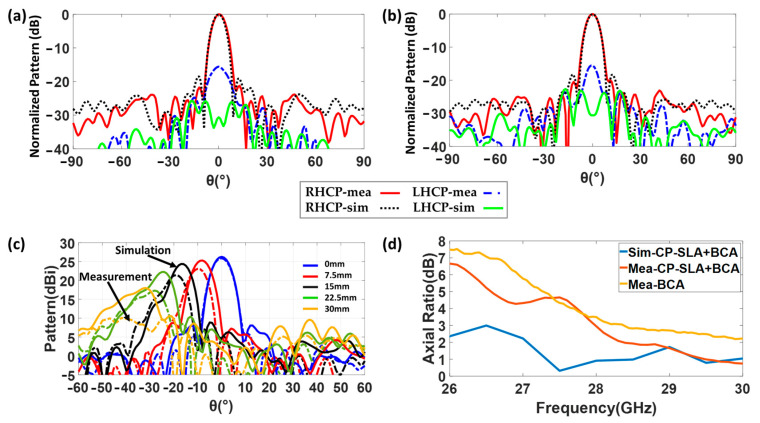
(**a**) Measured and simulated normalized radiation pattern at 28 GHz (ϕ=0°). (**b**) Measured and simulated normalized radiation pattern at 28 GHz (ϕ=90°). (**c**) Measured and simulated radiation pattern (directivity) of the structure at different positions (ϕ=90°). (**d**) Measured and simulated axial ratio of CP-SLA + BCA.

The simulated (with losses) and measured normalized radiation patterns of the structure at 28 GHz are presented in Figure 18a,b, for H-plane (XZ, ϕ=0°) and E-plane (YZ, ϕ=90°), respectively. The RHCP results for measurements and simulations in both planes are in good agreement, though some null filling between main and side lobes in both planes can be seen. The LHCP results show a difference of about 15 dB at θ=0°.

As for the LP-FLA + PHA prototype, the BCA feeder has been tested in several x-offset positions (similar to Figure 14c) along the H-plane to test its scanning capability. CP-SLA was moved using two parallel rows of holes that are made on the fiber glass plate for allowing this displacement (see Figure 16). This structure provides a pointing angle range from −35° to 35° (±15° more than the coverage of the LP-FLA + PHA), with a directivity higher than 10 dBi. The maximum directivity (26 dBi) and minimum side lobe level (SLL) are attained when the antenna is positioned to align the center of the lens antenna in broadside. The lower measured directivity of 10 dBi and the highest side lobe level (SLL) are reached when the antenna is placed 30 mm (steering angle of 35°) apart from the center of the lens and the focal point (Figure 18c). The drop of directivity in the steered beam is unavoidable as the aperture efficiency reduces because of the aperture E-field phase shift. In addition, during measurement, misplacement of the feeder and supporters, and fabrication errors, can lead to additional degradations as well.

Due to the stepped lens symmetry, it is possible to place a number of feeders in such a way that they all provide separate beams, leading to a MIMO high directional symmetric 2D coverage. The axial ratio of the structure has also been investigated in Figure 18d. The circular polarization is available in whole bandwidth in simulation. In measurement, the axial ratio below 3 dB is available from 28 GHz to 30 GHz. This degradation is caused by misalignment and a frequency shift coming from the feeder. The BCA was manufactured using a 3D printer and then copper coated using an in-house, non-professional procedure. Thus, misalignment was unavoidable. In fact, the copper coating procedure was used multiple times, which caused the antenna thickness to expand by around 0.1 mm. Thus, the interior dimension of the antenna decreased below the nominal value due to the additional thickness and a frequency shift to a higher value is consequently seen.

## 5. Comparison

In this last section, a comparison of the discussed structures is presented for a better understanding of the designed antennas. This comparison has been carried out in two stages. First, the parameters related to the LP-FLA + PHA and the CP-SLA + BCA systems are collected in Table 3. Second, both presented systems have been compared in terms of performance with similar structures, which have already been presented in previous sections.

At this point, it is essential to clarify that the maximum theoretical directivity for both cases (26.8 dBi) has been determined with an ideal 100% total aperture efficiency ($\eta_{Total-aper})$ in (2), and the same lens aperture area ($A_{lens-aper}=\pi D^{2}/4$, with D=75 mm for the lens diameter) using the method explained in [27]. In addition, the measured total aperture efficiency has been determined through $D_{0}={D_{max}\cdot \eta }_{Total-aper}$, using the measured directivity of the two prototypes, which are included in Table 3. According to the given information, it is clear that a better performance is achieved for the CP-SLA novel design in terms of measured directivity (26 dBi versus 24.6 dBi) and total aperture efficiency (82% versus 60%). This is explained by its better phase uniformity and frequency stability compared to the LP-FLA. Nevertheless, as the total substrate volume is larger in the stepped CP-SLA configuration, higher material losses (2 dB versus 1.1 dB) and consequently lower radiation efficiency are obtained (60% versus 72%). The advantage of the novel CP-SLA is finally verified in terms of the realized gain, with a 23 dBi to 24.3 dBi variation from 26 GHz to 30 GHz. The LP-FLA remains almost constant around 23 dBi in the same frequency range. This improved performance has been achieved with a significant antenna volume reduction in the CP-SLA + BCA prototype thanks to the smaller focal distance. These findings imply that it is feasible to enhance the radiation efficiency by reducing the loss with a material with a lesser loss tangent at the expense of a higher cost.

A comparison with other works is detailed in Table 4. Reference [27] presented a perforated planar lens that is fed by a stacked-patch microstrip antenna with four input ports. Although the lens size is smaller than in our work, the maximum measured gain is obviously less. The work in [29] uses a perforated planar lens made by milling the dielectric disk, which is closer to the fabrication described here. Although the feeding involves an open waveguide that is mechanically moved to create the guided beam, the experimental performance is good. The complete volume for the structure is small, and the gain is less than 20 dBi, which is lower than in the presented works. A new type of GRIN metamaterial lens is introduced in [30], with a maximum realized gain of around 21.5 dBi. Although the total size of the lens antenna is smaller, the aperture efficiency is lower than in the presented works. A perforated planar lens [4] has been compared with the presented works. The size of the lens is smaller, while the maximum realized gain is lower. In [31], a significantly bulky spherical perforated lens is introduced that provide an aperture efficiency of 67%. In conclusion, the CP-SLA + BCA and the LP-FLA + PHA have reasonable size with better performance in comparison with previous works. Moreover, when it comes to aperture efficiency, the presented prototypes overperform previous works.

## 6. Conclusions

Two different types of perforated GRIN lens antennas have been designed and manufactured using an inexpensive 3D printing technique. By locating the proposed lens antennas in front of two types of feeders, the LP-FLA + PHA and the CP-SLA + BCA systems are developed to confirm their application for 5G communications at the 26 GHz–30 GHz frequency range. The diameter of both lens antennas is 75 mm, but their focal distances vary due to their different feeders. For the LP-FLA, the feed antenna is a typical pyramidal horn antenna (PHA) utilized for terrestrial applications. For CP-SLA, the feeder is a novel bow-tie cavity antenna (BCA) providing circular polarization, which makes the system suitable for satellite communications. In the CP-SLA + BCA, the smaller focal distance (24.6 mm) reduces the total volume of the structure in comparison with the LP-FLA + PHA (64 mm).

To validate the obtained performances, related experimental investigations were carried out. It has been shown that the highest directivity in both cases was the same during the design process. However, for the measured results, the LP-FLA + PHA obtained 24.6 dBi, and the CP-SLA + BCA achieved a significantly closer result of 26 dBi to the required theoretical directivity of 26.8 dBi at the center frequency. The resemblance of measured directivity to simulated directivity in the CP-SLA + BCA is related to the improved phase uniformity of the lens at the aperture, consequence of the stepped shape of the lens design. Although the thicker lens property causes more loss in the CP-SLA, the measured realized gain in this unique design is greater in the LP-FLA. Nevertheless, both structures produce a worthwhile outcome.

Total aperture efficiency of 82% and 60%, in the CP-SLA + BCA and the LP-FLA + PHA, respectively, have been attained with the mentioned design method, validating the concept, and demonstrating outstanding performance in comparison to previous works. Both the CP-SLA + BCA and the LP-FLA + PHA systems achieve a radiation beam steering of 20° (with a directivity over 15 dBi) and 35° (with a directivity over 10 dBi), respectively.

## Figures and Tables

**Table 1:** Parameters of the planar graded-index lens.

Ring (*i*)	$\rho_{ext}\;(mm)$	$\rho_{int}\;(mm)$	No. of Holes	${ɛ}_{ri,eff}$
0	2.75	0	0	2.5
1	9.5	2.75	10	2.241
2	14.5	9.5	18	2.244
3	19.25	14.5	34	2.18
4	23.4	19.25	48	2.07
5	26.25	23.4	66	1.99
6	29.7	26.25	76	1.75
7	32.25	29.7	80	1.70
8	34.65	32.25	82	1.69
9	37.5	34.65	88	1.82

**Table 2:** Permittivity parameters of the stepped graded-index lens.

Ring (*i*)	$\rho_{ext}\;(mm)$	$\rho_{int}\;(mm)$	No. of Holes	Height	${ɛ}_{ri,eff}$
0	3.75	0	0	40	2.5
1	7.5	3.75	5	40	2.23
2	11.25	7.5	6	40	2.2
3	15	11.25	10	40	2.17
4	18.75	15	16	40	2.1
5	22.5	18.75	21	37	2.05
6	26.25	22.5	27	34	2
7	30	26.25	41	31	1.85
8	33.75	30	53	28	1.75
9	37.5	33.75	67	25	1.65

**Table 3:** Comparison of LP-FLA and CP-SLA directivity and total aperture efficiency at 28 GHz.

	Directivity-Mea	Directivity-Max (Theoretical)	Total Aper. Efficiency (%)-Mea	Feeder Loss-(dB)	Material Loss (Lens)-(dB)	Realized Gain-Mea	Rad-Efficiency (%)-Mea
**LP-FLA**	24.6	26.8	60	0.3	1.1	23.2	72
**CP-SLA**	26	26.8	82	0.2	2	23.8	60

**Table 4:** Comparison of LP-FLA and CP-SLA with similar works.

	Ref. [27]	Ref. [29]	Ref. [30]	Ref. [4]	Ref. [31]	LP-FLA	CP-SLA
**Design Frequency (** GHz **)**	26	60	9.5	28	28	28	28
**Lens Type**	Perforated Planar	Perforated Planar	Metamaterial lens	Perforated Planar	Perforated spherical	Perforated Planar	Perforated Stepped
**Lens diameter**	6.7 λ_0_	6 λ_0_	4.56 λ_0_	3.2 λ_0_	10 λ_0_	7 λ_0_	7 λ_0_
**Lens Thickness**	2.2 λ_0_	1.4 λ_0_	0.38 λ_0_	0.7 λ_0_	7.3 λ_0_	2.3 λ_0_	2.3–3.7 λ_0_
**Lens feeder Spacing**	4.5 λ_0_	1.5 λ_0_	3.2 λ_0_	5.9 λ_0_	0.22 λ_0_	5.9 λ_0_	2.3 λ_0_
**Max measured Realized gain (** dBi **)**	20.2	18.3	21.5	20.7	21.2	23.6	24.3
**Feeder**	Multi-port	WR-15	antipodal exponential taper slot antenna	microstrip patch antenna	Phased Array antenna	Horn	Bow-tie antenna
**Total Aper. efficiency (%)**	38	N.A	50	N.A	67	60	82

## Data Availability

No new data were created or analyzed in this study.
